# Molecular Insights Into Therapeutic Potential of Autophagy Modulation by Natural Products for Cancer Stem Cells

**DOI:** 10.3389/fcell.2020.00283

**Published:** 2020-04-24

**Authors:** Md. Ataur Rahman, Subbroto Kumar Saha, Md Saidur Rahman, Md Jamal Uddin, Md. Sahab Uddin, Myung-Geol Pang, Hyewhon Rhim, Ssang-Goo Cho

**Affiliations:** ^1^Center for Neuroscience, Korea Institute of Science and Technology, Seoul, South Korea; ^2^Department of Biotechnology and Genetic Engineering, Global Biotechnology & Biomedical Research Network, Islamic University, Kushtia, Bangladesh; ^3^Department of Stem Cell and Regenerative Biotechnology, Konkuk University, Seoul, South Korea; ^4^Department of Gynecology and Obstetrics, Johns Hopkins School of Medicine, Baltimore, MD, United States; ^5^Department of Animal Science & Technology and BET Research Institute, Chung-Ang University, Anseong, South Korea; ^6^Graduate School of Pharmaceutical Sciences, College of Pharmacy, Ewha Womans University, Seoul, South Korea; ^7^ABEx Bio-Research Center, Dhaka, Bangladesh; ^8^Department of Pharmacy, Southeast University, Dhaka, Bangladesh; ^9^Pharmakon Neuroscience Research Network, Dhaka, Bangladesh; ^10^Division of Bio-Medical Science and Technology, KIST School, Korea University of Science and Technology, Seoul, South Korea

**Keywords:** autophagy, cancer stem cell, natural products, stemness, chemoresistance

## Abstract

Autophagy, a cellular self-digestion process that is activated in response to stress, has a functional role in tumor formation and progression. Cancer stem cells (CSCs) accounting for a minor proportion of total cancer cells-have distinct self-renewal and differentiation abilities and promote metastasis. Researchers have shown that a numeral number of natural products using traditional experimental methods have been revealed to target CSCs. However, the specific role of autophagy with respect to CSCs and tumorigenesis using natural products are still unknown. Currently, CSCs are considered to be one of the causative reasons underlying the failure of anticancer treatment as a result of tumor recurrence, metastasis, and chemo- or radio-resistance. Autophagy may play a dual role in CSC-related resistance to anticancer treatment; it is responsible for cell fate determination and the targeted degradation of transcription factors via growth arrest. It has been established that autophagy promotes drug resistance, dormancy, and stemness and maintenance of CSCs. Surprisingly, numerous studies have also suggested that autophagy can facilitate the loss of stemness in CSCs. Here, we review current progress in research related to the multifaceted connections between autophagy modulation and CSCs control using natural products. Overall, we emphasize the importance of understanding the role of autophagy in the maintenance of different CSCs and implications of this connection for the development of new strategies for cancer treatment targeting natural products.

## Introduction

**FIGURE 1:**
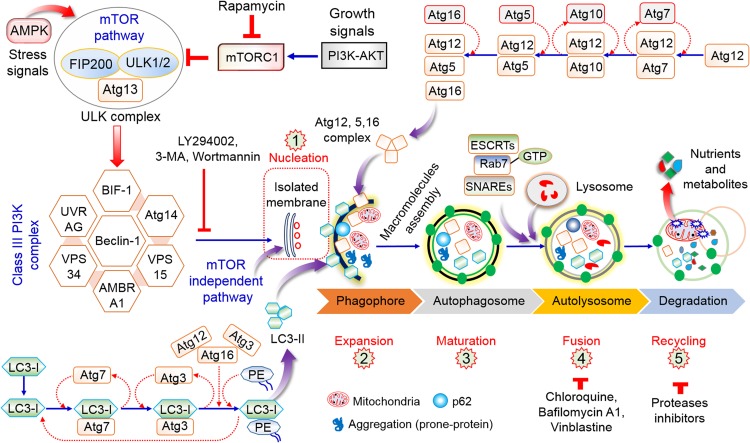
Role and regulation of the overall autophagy process. The initiation of autophagy is dependent on nutrient deprivation or growth factors that activate AMPK or inhibit mTORC1, thus stimulating the ULK1 complex (ATG13 and FIP200). The phosphorylation of Beclin-1 leads to the activation of VPS34, which stimulates phagophore initiation and formation. Atg5–Atg12 conjugation stimulates Atg7 and Atg10 to form the Atg12–Atg5–Atg16 complex, which ultimately influences phagophore formation. The Atg7, Atg3, and Atg16 complex has a function similar to that of E3 in the direction of the LC3-PE association (LC3-II) for formation of the autophagosome. The maturation of the autophagosome involves fusion to lysosomes via ESCRT, SNARE, and Rab7. Lysosome binds to autophagosome by several lysosomal proteins leading to cargo degradation as well as metabolite production and recycling of nutrient products (Rahman and Rhim, 2017).

Autophagy is an intra-cellular molecular mechanism and pathway for self-digestion, in which unwanted cytoplasmic components, such as proteins, toxic compounds, injured/damaged organelles, lipid molecules, and mitochondria, are sequestered into membrane-bound vesicles (phagophore), which eventually form autophagosomes in order to recycle and degrade these substances (Rahman and Rhim, 2017). In normal conditions, autophagy is essential for the conservation of the overall cellular homeostasis, and contributing to protein and organelle quality control (Uddin et al., 2018). It also contributes to the stress response (for example, in conditions like hypoxia, chemical exposure, and starvation). The initiation and regulation of autophagy signaling are described in Figure 1. Recent research has emphasized the important role of autophagy in cancer cell adaptability, growth, and survival in the tumor microenvironment (Lei et al., 2017). Autophagy has dual roles in cancer cells, including tumor-stimulating as well as tumor-suppressive functions (Avalos et al., 2014), with a wide range of effects on tumor progression and oncogenesis (Avalos et al., 2014). Recent research has clearly established that autophagy is an important target for the control and management of cancer. However, the control factors depend on the stage and type of tumor, host microenvironment, systemic cellular location, and management strategy (Ojha et al., 2015; Sohn et al., 2017). Overall, autophagy has both stimulatory and suppressive effects on cancer and cancer stem cells (CSCs) and contributes to metastasis.

**FIGURE 2:**
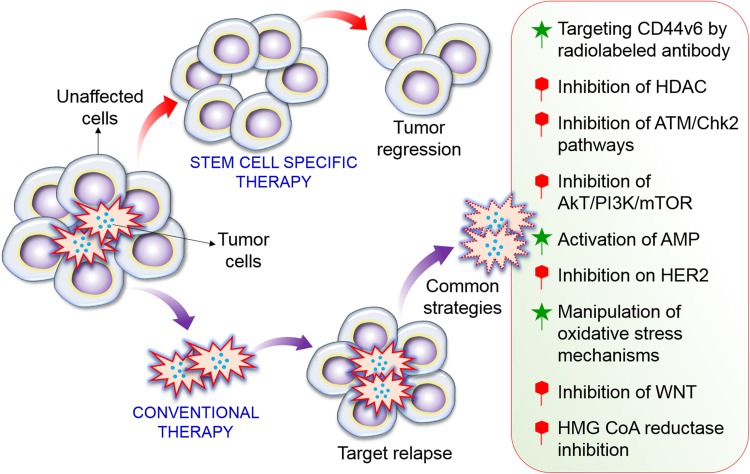
Formation of CSC and precise targets of CSC pathways. A high dose of radiotherapy has the potential to cure solid tumors via the inactivation of all CSCs. Recurrence can generally be attributed to one or a few remaining CSCs. A combined treatment strategy that precisely targets CSCs or several key molecular signaling pathways in CSC processes to control tumor cell growth would be expected to improve local tumor control. Among of them inhibition of HDAC, Akt/mTOR, HER2, WNT, and HMG CoA reductase inhibition is important to regulate CSCs. While activation of AMPK, manipulation of oxidative stress, and targeting CD44v6 also important of CSC pathways.

Cancer stem cells exhibit features of normal stem cells, including self-renewal, differentiation, and tumorigenicity. Tumor formation and recurrence might be mainly be regulated by CSCs (Figure 2) (Gupta et al., 2009a; Suresh et al., 2016). Therefore, advances in anticancer therapies require the elucidation of the mechanisms underlying the survival and stemness of CSCs. Targeted therapy is a major strategy to reprogram CSCs into normal stem cells (Eun et al., 2017; Saha et al., 2017, 2018). In particular, targeting autophagy is a potential strategy to regulate CSCs (Nazio et al., 2019; Smith and Macleod, 2019). Although stimulation of autophagy exhibits side effects in some chemotherapies (Apel et al., 2008; Liu et al., 2011), autophagy has a useful and valuable therapeutic function in many cancers by mediating initiation of immunogenic cell death and clearance (Galluzzi et al., 2017). Therefore, to exploit autophagy stimulation and suppression for cancer prevention and management, it is essential to consider the precise role of this process in individual types of cancer and to determine the efficacy of autophagy regulation in cancer therapy. However, the critical mechanisms underlying CSC maintenance and functions with respect to autophagy have not been clearly described. This review emphasizes the role of autophagy in CSC biology and considers how targeting this process might impair CSC formation and survival. Furthermore, we discuss how autophagy contributes to each step of CSC physiology, including differentiation, generation, invasion, and migration, as well as pharmacological control by natural products.

## Characteristics of Cancer Stem Cells

Cancer stem cells exhibit self-renewal and can differentiate into various cell types, including tumor cells (Mackillop et al., 1983). These cells are a small population of cancer cells that are responsible for tumor growth and heterogeneity, and contribute to metastatic activity and resistance against conventional anticancer therapies (based on *in vivo* or *in vitro* analyses) (Lobo et al., 2007). CSCs have been identified as subpopulations of acute myeloid leukemia (AML) cells that express CD34, a specific surface marker. Though initially recognized in AML, CSCs have since been detected in various solid and difficult-to-treat cancers, such as pancreatic, brain, ovarian, colon, lung, melanoma, and breast cancers (Singh et al., 2004; Hermann et al., 2007; Li et al., 2007; O’Brien et al., 2007; Ricci-Vitiani et al., 2007; Eramo et al., 2008; Schatton et al., 2008; Zhang et al., 2008; Boiko et al., 2010). Importantly, CSCs are likely involved in tumor growth, with astonishing self-renewal and differentiation abilities that give rise to diverse cell phenotypes. They are characterized by the presence of particular cell surface markers, which could be used to differentiate these cells from normal and other tumor-forming cells. Therefore, these markers provide a basis for the establishment of several *in vitro* as well as *in vivo* approaches to separate, manipulate, and control CSCs. Additional essential characteristics of CSCs can explain unusual malignancies in an immune-deficient mouse model (Lobo et al., 2007). Breast cancer is a well-described human solid and condense tumor comprised of various resident cells, including CSCs and non-CSCs. The subpopulation of CSCs (CD44^+^ and CD24^–^/low) has been detected in the early stages of tumor progression in mice deficient in immune response factors (Al-Hajj et al., 2003). However, the lack of success of traditional treatment strategies is closely associated with the plasticity of CSCs due to their unrestricted self-renewal and differentiation characteristics, potential proliferative activity, and ability to inactivate components of the cell pool. An understanding of the molecular and cellular mechanisms underlying CSC proliferation and survival remains critical for expanding the usefulness of current therapeutic approaches.

**FIGURE 3:**
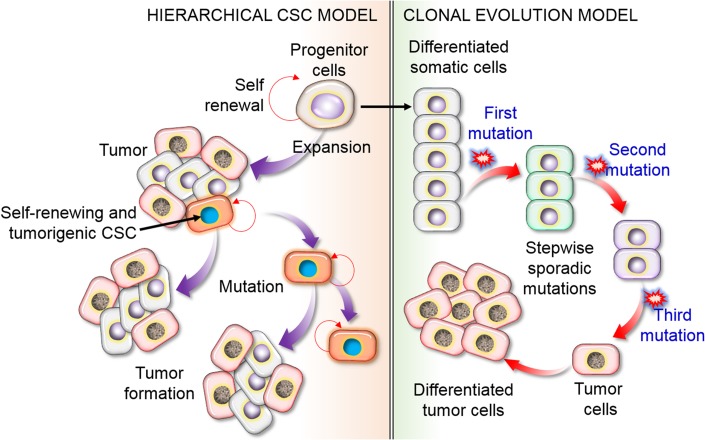
Schematic representation of the hierarchical CSC model of CSCs versus the clonal evolution or stochastic model of tumor cell heterogeneity. The hierarchical model proposes that only limited subpopulations of CSCs have the ability to initiate the development of cancer, with particular (intrinsic) features that could be recognized and targeted to destroy a tumor. In the stochastic model, to form cancerous cells, it is necessary to undergo a substantial series of DNA modifications. In this process, stepwise mutation causes tumor cells. Mutations could happen in any cell, resulting in cancer formation. This concept fundamentally suggests that all cells have the capacity to be tumorigenic with self-renewal or differentiation ability, leading to tumor heterogeneity, and other cells are differentiated as non-CSCs.

Two key models have been proposed to explain the tumor cell source and heterogeneity. According to the stochastic model, all cancer cells can induce new tumors cells by transforming from non-CSCs to the CSC phenotype via an energetic mechanism in response to particular stimuli, such as mutations. The second model is the hierarchical model, in which a single group of CSCs contributes to tumor occurrence and increases heterogeneity by producing differentiated and inactive cancer cells (Figure 3). While these phenotypes and models appear to be mutually exclusive, it is possible that a combination of the two models explains the observed patterns.

## Maintenance and Survival of Cancer Stem Cells by Autophagy

**FIGURE 4:**
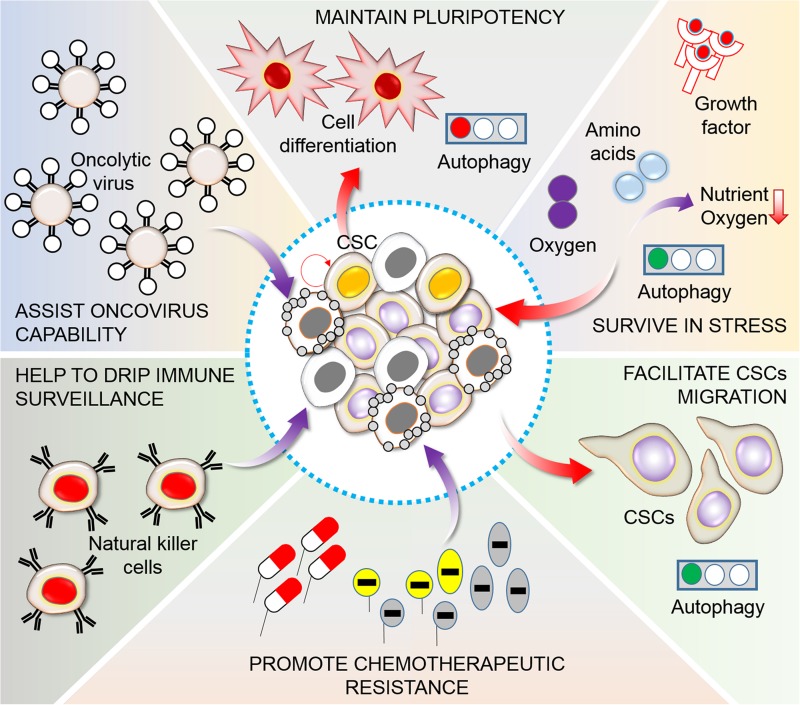
Functions of autophagy in CSC maintenance. A heterogeneous population of tumor cells generally consists of a small CSC population. CSCs are frequently related to an elevation of autophagy levels, which sustains pluripotency, promotes survival in limited nutrient conditions and hypoxia in the tumor microenvironment, controls migration and invasion, and stimulates chemotherapy resistance, escape from immunosurveillance of natural killer (NK) cells, and maintenance oncovirus competence (Nazio et al., 2019). In this consequence, manipulation of autophagy is found to be essential for targeting of cancer cells. Generally, during this pathway autophagy act in addition to contribute CSC differentiation, plasticity, generation, physiology, migration and invasion, viral and immune-resistance, and pharmacological properties.

The maintenance and aggressiveness of CSCs are fundamentally related to autophagy. CSCs are characterized by their self-renewal capacity and differentiation ability as compared to normal stem cells (Lobo et al., 2007). However, pluripotency is a fundamental characteristic of CSCs that permits unlimited growth and division as well as the maintenance of undifferentiated cells. The manipulation of autophagy is essential for the regulation of cancer cells and CSCs (Figure 4). CSCs were first identified in AML based on the expression of the cell surface markers, CD34 and CD38 (CD34^+^ and CD38^–^), using fluorescence-activated cell sorting (Zhang et al., 2008). However, studies on breast CSCs have shown that autophagy maintenance and homeostasis are fundamental requirements for the conservation of pluripotency in several pathophysiological conditions (Han et al., 2018; Nazio et al., 2019). Autophagy is enhanced in mammospheres and is correlated with adherent cells expressing Beclin-1 as well as Atg4. These two important autophagy proteins are required for the conservation, maintenance, and expansion of cells (Gong et al., 2012). Recent studies have demonstrated that autophagy is associated with CSCs in various types of cancer, including liver, pancreatic, breast, ovarian, glioblastoma, and osteosarcoma (Chaterjee and van Golen, 2011; Gong et al., 2013; Song et al., 2013; Zhang et al., 2016; Peng et al., 2017; Buccarelli et al., 2018). However, in hematological malignancies, autophagy can act as a tumor-suppressive or chemoresistance factor. Thus, depending on the kind of progenitors as well as the disease condition, autophagy might have opposite functions. Several autophagy-related genes (*Beclin-1*, *Agt4*, and *Atg5*) are upregulated, and the silencing of these genes negatively affects cell survival; as a result, the extent of autophagy in chronic myeloid leukemia (CML) appears to be related to the status of CSCs in the tumor (Rothe et al., 2014; Karvela et al., 2016). In contrast, efficient autophagy is necessary to protect against the progression of myelodysplastic syndrome into AML, and several additional autophagy-related genes (Atgs) are mutated or deregulated in patients with AML (Houwerzijl et al., 2009). Alternatively, blockage of autophagy leads to reduction in the levels of TFGβ2 and TGFβ3, thereby inhibiting the Smad pathway that is crucial for the CD29hiCD61^+^ CSC phenotype. Moreover, inhibition of autophagy downregulates IL-6 secretion, possibly via the JAK2/STAT3 signaling, in triple-negative autophagy-dependent breast cancer stem cells (BCSCs) (Maycotte et al., 2015). The secretion of IL-6 is an important factor for CSC conservation as well as maintenance, and promotes the CD44^+^/CD24 low phenotype in breast cancer (Iliopoulos et al., 2011). The IL-6-JAK2-STAT3 pathway might play an essential role in the transformation from non-CSCs to CSCs.

Additional studies have proposed a role for the Forkhead box protein O (FOXO) in controlling the fate of CSCs (van Doeselaar and Burgering, 2018). FOXO-mediated transcriptional regulation is essential for homeostasis of stem cells in embryos as well as in adult cells (Gargini et al., 2015). However, it is important to distinguish how FOXO transcriptional activity contributes to CSC functions and maintenance. The silencing of FOXO3 improves the CSC renewal ability in breast, ovarian, colorectal, prostate, and liver cancer, and in glioblastoma cells (Dubrovska et al., 2009; Ning et al., 2014; Prabhu et al., 2015; Smit et al., 2016). Leukemia-initiating cells require FOXO3 for the maintenance of stem cell homeostasis (Pellicano et al., 2014). Conversely, from the perspective of autophagy, FOXOs regulate various Atgs, such as Beclin-1, LC3, ULK1, ATG5, ATG8, GABARAPL1, ATG12, ATG14, and BNIP3 (van Doeselaar and Burgering, 2018), and cytosolic FOXOs contribute to autophagy control and regulation. Most recently, it has been shown that AMBRA1, a pro-autophagic protein, is fundamental for controlling T-cell differentiation and for maintaining homeostasis, regulating the FOXO3–FOXOP3 interaction. Therefore, this might be involved in the FOXO3-dependent autophagy modulation in various cellular processes (Becher et al., 2018). Additional studies are needed to precisely determine how the FOXO-mediated control of stemness as well as autophagy are interrelated in tumorigenesis. Interestingly, recent studies have suggested that there is crosstalk between autophagy and staminal markers as well as the biosynthetic pathway of NAD^+^. Inefficiencies in normal autophagy created by autophagy inducers as well as inhibitors decrease the pluripotency of CSCs in teratocarcinoma, promoting differentiation in addition to cellular senescence (Ali Azouaou et al., 2015). Overall, accumulating evidence supports the complexity of the autophagy-mediated modulation of CSCs. A unique relationship between autophagy and stemness has recently been identified in ovarian cancer stem cells (OCSCs) (Peng et al., 2017). The overexpression of Forkhead Box A2 (FOXA2) in OCSCs is regulated through the autophagy pathway. The blockage or inhibition of autophagy via both pharmacological and genetic procedures promotes FOXA2 downregulation and the subsequent impairment of the self-renewal ability of cells (Peng et al., 2017). Another study has identified a pathway for autophagy regulation and the control of chromosome immovability by synchronizing the ATR checkpoint and the double-strand-break pathway (Robert et al., 2011). Therefore, CSCs might exploit autophagy to inhibit additional DNA damage and thereby improve survival.

## Autophagy-Induced Resistance of Cancer Stem Cells to Chemotherapy

**FIGURE 5:**
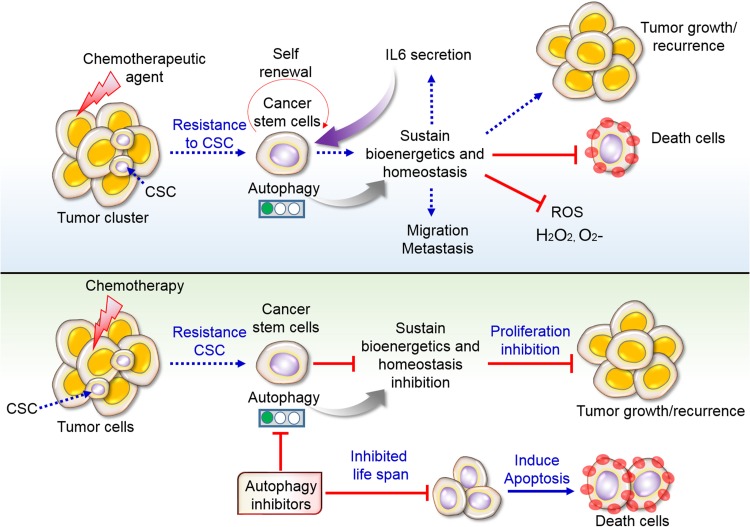
Resistance of CSCs to chemotherapy via autophagy. Conventional chemotherapy disrupts cell growth; however, CSCs remain unaffected by chemotherapy and sustain autophagy-mediated survival. Sustain cells further recurrence and make tumor heterogeneity along with migration and metastasis. Some cells become death due to therapeutic agents. Secretion of interleukin 6 (IL6) stimulates cancer cells self-renewal and proliferation. Inhibition of autophagy prevents cellular life span in addition to influence apoptotic cell death by chemotherapy. For that reason, targeting autophagy inhibitors in tumors and CSCs might overcome the resistance as well as inhibit tumor growth.

Diverse molecular mechanisms contribute to CSC resistance to drug treatment, including cellular plasticity, highly efficient DNA damage repair, expression of genes related to multi-drug resistance, and prevention of apoptosis. A correlation between CSCs and drug resistance has also been observed in numerous human cancers, including breast, pancreatic, melanoma, leukemia, colorectal, and brain cancers (Abdullah and Chow, 2013). Among various cellular and molecular mechanisms involved in resistance to chemotherapeutics, autophagy appears to be critical (Ojha et al., 2015) (Figure 5). Furthermore, it has been well-documented that chemotherapeutic treatments are intrinsically able to prompt autophagy in cancer cells (Sui et al., 2013). Numerous studies using various investigational methods have shown that combinations of cytotoxic agents and autophagy mediators enhance CSC sensitivity. In neuronal glioblastoma (GBM) stem cells, an EGFR inhibitor [bevacizumab or temozolomide (TMZ)] combined with a late-stage autophagy blocker, chloroquine (CQ), improves drug toxicity, leading to the impairment of GBM CSC proliferation and survival (Golden et al., 2014; Huang et al., 2018). In gastric CSCs, CQ and 5-fluorouracil block Notch signaling and decrease viability (Li L. Q. et al., 2018). However, JAK-induced autophagy is involved in progression in cisplatin-resistant bladder cancer cells (Ojha et al., 2016b). In AML CSCs or glioma stem cells (GSCs), ATG7 knockdown potentiates the inhibitory effect of salinomycin on cell proliferation and existence (Yue et al., 2013). In particular, a combination of autophagy blockers, bafilomycin A1 or CQ, with tyrosine kinase inhibitors (TKIs) disrupts CML cell proliferation and existence (Bellodi et al., 2009). Additional studies have indicated that autophagy might function in drug-induced cytotoxicity (Rahman et al., 2016, 2017). Resveratrol, a polyphenolic compound, affects breast CSC growth and survival by blocking Wnt signaling, which promotes autophagy (Rahman et al., 2012a; Fu et al., 2014). The inhibition of mTOR promotes neuroblastoma as well as the differentiation of glioma CSCs (Zeng and Zhou, 2008; Zhao et al., 2010; Rahman et al., 2016, 2017). Overall, the specific involvement of autophagy in the drug resistance of CSCs provides a basis for the development of different antineoplastic therapies.

## Role of Autophagy in the Control of Different Cancer Stem Cells With Natural Products

**FIGURE 6:**
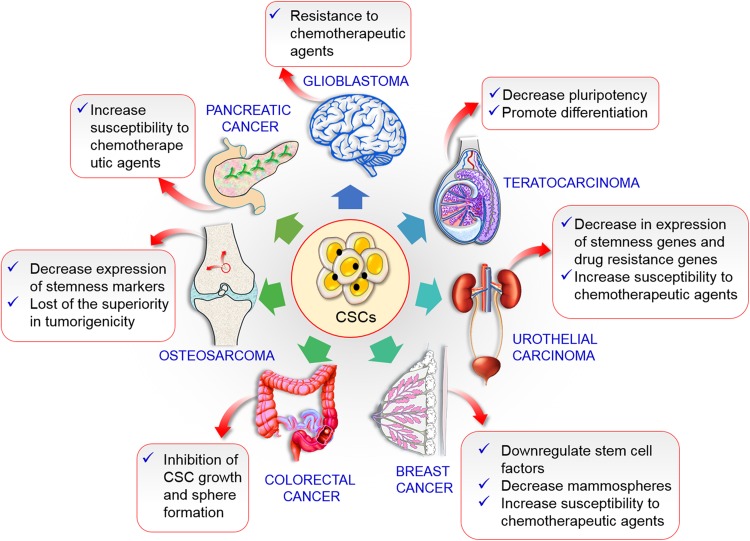
Prospective outcomes inhibition of autophagy various CSCs. In breast cancer decreases stem cell factors and mammospheres along with increases chemotherapeutic agents susceptibility. Inhibition of autophagy colorectal CSC causes growth inhibition. Tumorigenicity was lost in osteosarcoma CSC. Increase susceptibility to chemotherapies in pancreatic and urothelial CSCs autophagy inhibition. Autophagy reduction cause glioblastoma cells chemotherapies resistance.

As both autophagy and CSCs are critical modulators in resistance to anticancer therapy, it is important to explore their correlation. A combination of gemcitabine (GC) and autophagy inhibitors (e.g., CQ) augments the propensity of pancreatic CSCs (Yang et al., 2015). A reduction of autophagy may hinder the preservation of CSCs. Salinomycin is more efficient than paclitaxel for reducing breast CSCs (Yue et al., 2013). Blocking autophagy by CQ sensitizes triple-negative breast cancer (TNBC) cells to paclitaxel by the modulation of JAK2 and DNMT1, thereby decreasing the levels of CD44^+^/CD24^–^/low CSCs. Autophagy also induces resistance to photodynamic therapy in CSCs in colorectal cancer (Wei et al., 2014). As a result, autophagy is expected to increase the efficacy of anticancer therapy. It is a prospective target for shifting anticancer treatment resistance in CSCs. The outcome of the inhibition of autophagy in CSCs is summarized in Figure 6. Additionally, mechanism of various natural compound in different CSCs regulation in relation to autophagy are presented in Table 1 and summarized the following points.

**TABLE 1:** Natural products that modulates autophagy targeting cancer stem cells.

Natural products	Autophagy/mechanism	Cancer stem cells	References
Everolimus	PI3K/mTOR inhibitor	Breast cancer stem cells	Cavazzoni et al. (2012)
Salinomycin	Autophagy induction	Breast cancer stem cells	Zhao et al. (2019)
18α-Glycyrrhetinic acid and Gambogic acid	Induction/mTOR inhibition	Neuroblastoma brain cancer cells	Rahman et al. (2013, 2016)
Curcumin	Autophagy induction	Colon cancer stem cells	Huang et al. (2016b)
Resveratrol	Induction/mTOR inhibition	Colon cancer stem cells	Miki et al. (2012)
Rottlerin	PI3K/Akt/mTOR inhibition	Pancreatic CSCs	Singh et al. (2012)
Apigenin	PI3K/Akt/mTOR inhibition	CD34^+^ and CD38^–^ leukemia cells	Cheong et al. (2010)
Alisertib	Akt/mTOR inhibition	Urinary bladder CSCs	Ding et al. (2015)
Trehalose	Autophagy induction	Prostate CSCs	Cristofani et al. (2018)
Gintonin	Induction/mTOR inhibitor	Neuroblastomas	Rahman et al. (2020a)

### Autophagy in Breast Cancer Stem Cells

The genetic inhibition of autophagy decreases the proportion of breast cancer cells with the CD44^+^/CD24^–^/low CSC-like phenotype, signifying a role of autophagy in maintaining typical CSCs in breast cancer (Cufi et al., 2011). Therefore, the inhibition of autophagic flux as well as lysosomal proteolytic function by K^+^/H^+^ ionophores by salinomycin efficiently decreases ALDH^+^ breast CSC population (Yue et al., 2013). It has also been established that the serum-starvation of mesenchymal stem cells (SD-MSCs) maintains MCF-7 tumor growth. SD-MSC tumors show high cellularity and differentiation as well as reduced apoptosis. Additionally, *in vitro* studies have revealed that SD-MSCs persist via autophagy, and the secretion of paracrine factors by these cells helps in the maintenance of tumor cells in nutrient/serum starvation conditions (Sanchez et al., 2011). However, Atg12, Atg5, and LC3B overexpression in quiescent stem cells has similar effects in breast cancer and the autophagy inhibitor 3-methyladenine restores the dormant phenotype (Chaterjee and van Golen, 2011). An increase in the expression of Beclin-1 in mammospheres has been detected in human breast cancer cells. Similar results have been obtained in additional breast cancer cell lines, such as BT474 and MCF-7. However, basal as well as a starvation-mediated autophagic fluxes are increased in aldehyde dehydrogenase 1-positive cells. These results confirmed that Beclin-1 is critical for CSC function and tumor growth, indicating that CSCs use autophagy for maintenance of growth and tumor persistence (Gong et al., 2013). Certainly, developed resistance to letrozole was decreased by treatment via a derivative of rapamycin RAD001, everolimus, and a dual PI3K/mTOR inhibitor, NVP-BEZ235, in breast cancer cells (Cavazzoni et al., 2012). Previously, it has been reported that FR122047 (FR), a known non-steroidal anti-inflammatory drug, can significantly induce the caspase-9 mediated cytoprotective autophagy but caspase7/8 mediated apoptosis in breast cancer cells (Jeong et al., 2011). However, salinomycin, a polyether ionophore antibiotic, eliminated BCSCs induction of lipid oxidation, mitochondrial dysfunction, and oxidative stress (Zhao et al., 2019). Thymoquinone, derived from *Nigella sativa*, increases the cytotoxicity in breast cancer cell lines via autophagy stimulation (Bashmail et al., 2018). Curcumin, natural phytochemical, preventing the proliferation of BCSCs along with decreasing the bulk breast cancer cells (Li and Zhang, 2014). Curcumin repressed BCSCs proliferation and migration through inhibiting β-catenin nuclear translocation as well as decreasing β-catenin expression transcriptional targets containing pro-EMT factors (Mukherjee et al., 2014). Resveratrol, polyphenols compound with anti-cancer activity, also repressed BCSCs mammospheres formation as well as tumorigenicity and facilitate autophagy by disrupting Wnt/β-catenin pathway (Fu et al., 2014). Genistein, isoflavone phytoestrogen dietary found in soybeans, repressed mammospheres formation through MCF-7 in addition to MDA-MB-231 cells moderately via decreasing PTEN/PI3K/Akt pathway (Montales et al., 2012). Quercetin, a dietary flavonoid from plants, could down-regulate P-glycoprotein and inhibit Y-box binding protein 1 nuclear translocation, thus improving chemosensitivity of breast cancer cells along with inhibiting BCSCs (Li S. Z. et al., 2018). 6-Shogaol, polyphenolic compounds derived ginger, disrupt Akt/GSK3β and hedgehog signaling pathway, thus mitigating stemness of BCSCs by autophagy (Wu et al., 2015).

### Autophagy in Colon Cancer Stem Cells

Colon cancer stem cells (CCSC) are chemo/radiotherapy-resistant and numerous CCSC markers with extracellular domains, such as DCLK1 (May et al., 2008), Lgr5 (Schepers et al., 2012), and CD44 (Park et al., 2012), have been identified. Immortalized embryonic epithelial cells overexpressing progastrin (HEKmGAS) exhibit improved metastatic/tumorigenic potential (Sarkar et al., 2012). However, non-tumorigenic (HEKC) cells do not express stem cell markers and transformed CSCs HEKmGAS coexpress DCLK1 and CD44. HCT-116, HT-29, and DLD-1 colorectal cancer cells coexpress DCLK1/CD44 stem cell markers, in a manner similar to that observed in HEKmGAS tumorigenic cells (Sarkar et al., 2012). It has been found that curcumin increases the persistence of CSCs in colon. Furthermore, optimal curcumin concentrations significantly decreased the levels of stem cell markers. Contrary to expectations, curcumin augments the proliferation and survival of autophagic CSCs. These results suggest that the cell survival benefits from autophagy, with implications for the long-term resolution of colorectal cancer (Kantara et al., 2014). Antroquinonol, isolation from mushroom *Antrodia camphorata*, triggered PI3K/Akt/β-catenin signaling and could be a favorable cancer prevention agent for colon CSCs (Lin et al., 2017). Bitter melon extracts, isolated from *Momordica charantia*, inhibit CCSCs by affecting energy homeostasis in addition to increase Beclin-1, Atg7 and 12 autophagic protein in HT-29, and SW480 colon cancer cells (Kwatra et al., 2013).

### Autophagy in Chronic Myeloid Leukemia Cancer Stem Cells

NSAID treatment increases LC3-II levels and decreases p62 levels, with a simultaneous decrease in the levels of numerous stemness-related markers, including Oct4, CD44, c-Myc, and mutant p53 in CD44^high^K562 cells, indicating that NSAIDs promote autophagy in CML cells (Moon et al., 2019). The dysregulation of autophagy-associated genes improves imatinib mesylate (IM)-induced cell death in cell lines as well as in primary CML cells. Combinatorial treatment with autophagy blockers and TKIs, such as IM, dasatinib, or nilotinib, results in the complete elimination of phenotypically as well as functionally distinct CML stem cells (Bellodi et al., 2009). It has previously been established that CML stem cells overexpressing p^210BCR/ABL^ are inherently resistant to Das, IM, bosutinib, and nilotinib (Copland et al., 2006; Konig et al., 2008). Therefore, alternative methods that combine a TKI with an additional agent are necessary for CML CSC therapy. Thus, autophagy inhibitors might improve the therapeutic effects of TKIs in CML. Recently, it has been showed that imatinib, a small molecule kinase inhibitor, facilitates K63-linked ULK1 ubiquitination subsequent in autophagy stimulation is critical for CML and perspectives for the treatment of CML CSCs treatment (Han et al., 2019). Resveratrol, a polyphenol compound, is an attractive agent that prompts autophagic cell death through preventing the AMPK/mTOR pathway in CML cells (Puissant and Auberger, 2010). Resveratrol also activated autophagic cell death in CML cells through together with AMPK and JNK-mediated p62/SQSTM1 expression (Banerji and Gibson, 2012). Metformin, isolated from *Galega officinalis*, treatment reduced the melanoma tumor growth of CML CSC in mice in addition to induce apoptosis and autophagy (Tomic et al., 2011).

### Autophagy in Brain Tumor Cancer Stem Cells

Glioblastoma is the most lethal tumor of the central nervous system (CNS) (Wen and Kesari, 2008). However, specific GBM types are highly distinct, with significant cellular heterogeneity, including minor subpopulations of GSCs. GSCs contribute to tumor initiation, malignant phenotypes, recurrence, and resistance to cancer therapy (Lathia et al., 2015). Higher levels of autophagy mesenchymal (MES) GSCs than that in proneural GSCs is related to increased resistance to therapy and tumorigenicity of MES GSCs (Huang et al., 2017). However, irradiation (IR) and chemotherapy in GBM, particularly with TMZ, increase autophagic activity (Kroemer, 2015; Liu et al., 2013). GBM uses autophagy as an escape machinery for cell survival in response to cytotoxic agents, including IR and TMZ, which are standard and effective first-line therapies (Kondo et al., 2005; White, 2015). However, the anti-glioma agent Delta-24-RGD promotes cell death by increasing autophagic protein expression as well as vacuole formation in brain stem cell lines derived from patients with GBM (Jiang et al., 2007). Delta-24-RGD-treated stem cells of brain tumor xenografts display enhanced persistence in a glioma-containing mouse model with higher levels of Atg5. Additionally, increasing data suggest that microRNAs (miRNAs) are dysregulated in cancer cells and that CSCs show different miRNA expression profiles in numerous types of tumors, including GBM. Context-dependent miRNA properties may control the response to therapy and tumorigenicity (Cascio et al., 2010; Liu et al., 2013; Huang et al., 2016a). Recently, it has been shown that miRNA-93, which is differentially expressed in MES and PN GBM subtypes, is related to autophagy in response to IR and TMZ (Huang et al., 2019). Therefore, the autophagy inhibitor CQ stimulates γIR-mediated cell death in extremely radioresistant patient-derived GSCs (Firat et al., 2012). Recently, study provided an understanding into the possible inhibitory mechanism of isatin, a plant compound, on neuroblastoma metastasis *in vivo* as well as *in vitro* (Cong et al., 2019). Natural products such as resveratrol, oxyresveratrol, aneglicin, gambogic acid, and 18α-Glycyrrhetinic acid induces apoptosis and autophagy in brain tumor neuroblastoma cells (Rahman et al., 2012a, 2013, 2016, 2017). Resveratrol expressively reduced TMZ resistance through downregulating NF-κB dependent signaling in T98G GBM cells (Huang et al., 2012) and activate AMPK pathway and mTOR signaling inhibition (Yuan et al., 2012). A bicyclic naphthoquinone plumbagin, found in the roots of Droseraceae, modulates numerous signaling pathways comprising Akt/mTOR, JNK, and NF-κB activates apoptosis and autophagy along with induces DNA damage as well as cell death in human brain tumor cells (Khaw et al., 2015). *Andrographis paniculata*, a medicinal herb cross the BBB, has been demonstrated that inhibits U87 as well as U251 GBM cell proliferation through inducing cell cycle arrest by reduced Cdk1 and Cdc25C expression and also displayed inhibition of PI3K/Akt/mTOR signaling pathway (Li et al., 2012). *Celastrus orbiculatus* extract was exposed to prevent cell proliferation, migration, and adhesion of human U87 and U251 GBM cells through PI3K/Akt/mTOR signaling pathway via autophagy signaling (Gu et al., 2016). Gintonin (GT), a glycolipoprotein isolated from the root of *Panax ginseng* Meyer, activates autophagy in a dose- and time-dependently manner via upregulated LC3-II expression in human U87MG GBM cells (Rahman et al., 2020a).

### Autophagy in Pancreatic Cancer Stem Cells

High rates of autophagy have been detected under basal conditions in pancreatic cancer cells (Yang et al., 2011; Endo et al., 2017); however, the relation between pancreatic CSCs and autophagy remains to be explored. Pancreatic CSCs are characterized by numerous putative markers, such as CD24, CD44, CD133, EpCAM, and ALDH1 (Li et al., 2007; Kim et al., 2011). In particular, the presence of CSCs in pancreatic cancer is related to poor outcomes (Rajeshkumar et al., 2010) as well as increased metastatic activity and chemoresistance (Rajeshkumar et al., 2010; Van den Broeck et al., 2012). Consequently, drugs that selectively target CSCs are promising treatments for pancreatic cancer. Zhu et al. (2013) found that HIF-1α as well as autophagy control contribute to the transition from pancreatic cancer non-stem cells to stem cells and further showed that high autophagic flux is related to enhanced HIF-1α expression. They suggested that a combination of HIF-1α and autophagy contributes to the regulation of equilibrium between CSCs and non-CSCs (Zhu et al., 2013). This observation highlighted the significance of therapeutic approaches directed at CSCs and the tumor microenvironment. The autophagy-mediated induction of OPN/NF-κB signaling is essential for pancreatic CSC maintenance (Yang et al., 2015), and the combination of GC and autophagy inhibitors is a favorable therapeutic approach for the control of pancreatic CSCs. Recently, some marine organisms could be used a favorable source to recognize novel pharmacologically dynamic substances to treat pancreatic CSCs via autophagy induction (Geisen et al., 2015). However, rottlerin, polyphenol natural product isolated from the Asian tree *Mallotus philippensis*, a protein kinase C-delta (PKC-δ) inhibitor, can encourage autophagic cell death in pancreatic CSCs (Singh et al., 2012). A bioactive constituent, thymoquinone, isolated from the volatile oil of the black seed *Nigella sativa*, triggered apoptosis as well as tumor growth prevention in pancreatic CSCs both *in vitro* and *in vivo* via Notch1 and PI3K/Akt/mTOR regulated autophagy signaling pathways (Mu et al., 2015).

### Autophagy in Urinary Bladder Cancer Stem Cells

Urinary bladder cancer, urothelial carcinoma (UC), is among the most common urogenital cancers worldwide (Witjes et al., 2014). The difficulty associated with the reversion of UC makes it amongst the most expensive human malignancies to manage and treat (Knowles and Hurst, 2015). CSCs are believed to be responsible for the initiation of UC as well as its progression and relapse (Ning et al., 2009). Autophagy signaling contributes to the survival response in bulk populations and in CSCs of urinary bladder cancer lines T24 and UM-UC-3 (Ojha et al., 2014). Hence, autophagy is related to cell survival in bladder carcinoma and is a potential target for the effective management of UC, and by extension to improved patient survival. Therefore, synergistic effects of GC and mitomycin (MM) cytotoxicity and the inhibition of autophagy potentially with a glycolytic inhibitor (2-deoxyglucose) might be helpful in improving the overall outcomes in patients with urinary bladder cancer (Ojha et al., 2016a). Alisertib, aurora kinase A inhibitor, returns balance of N-cadherin and E-cadherin which prevents phosphorylation of aurora kinase A as well as suppresses Akt/mTOR and induces G2/M phase arrest, autophagy, and apoptosis of urinary bladder CSCs (Ding et al., 2015). GC and MM are common DNA intercalating chemicals used to treat several UC types (Cheung-Ong et al., 2013). Additionally, both small interfering RNA and pharmacological autophagy inhibitors potentiate the effects of the chemotherapeutic agents, MM, GC, and cisplatin in urinary bladder CSCs from T24 and UM-UC-3 lines (Ojha et al., 2014). A bioactive polyphenol isolated from green tea, epigallocatechin-3-gallate (EGCG), repressed bladder cancer tumorspheres along with downregulated stem cell markers and inhibited proliferation-associated proteins and stimulated the apoptosis of bladder CSCs (Sun et al., 2019).

### Autophagy in Prostate Cancer Stem Cells

Prostate cancer is one of the important reasons of death in some cases of males. Natural plant extracts of Berberis libanotic Ehrenb has been found to reduce the capability of prostate CSCs originating from PC3, DU145, and 22Ru1 cells strongly downregulate prostate CSCs markers such as Oct4, Sox2, CD44, Nanog, and CD166. The invasion as well as migration abilities were expressively decreased in a concentration in addition to time-dependent mode (El-Merahbi et al., 2014). In fact, trehalose augmented LC3 in addition to p62 accumulation along with induction of LC3 puncta in prostate CSCs cells such as PC3, LNCaP, as well as DU145 (Cristofani et al., 2018). Koenimbin, a natural dietary constituent of Murraya koenigii (L) Spreng, suppressed of PC-3 cells in addition to target PC-3-derived CSCs via apoptotic as well as CSC signaling pathways in vitro (Kamalidehghan et al., 2018).

## Controlling Cancer Stem Cells Through Apoptosis Signaling Pathway With Natural Products

Program cell death or apoptosis is an acute cellular mechanism which facilitates survival as well as death via a multiple signaling transduction pathway (Elmore, 2007; Rahman et al., 2020b). Generally, the apoptotic pathway is damaged during cancer progression and development (Plati et al., 2011). However, apoptosis induction in CSC also require an additional attention for cancer therapy (Dragu et al., 2015). For that reason, numerous synthetic and natural compounds have been utilized to target extrinsic as well as intrinsic apoptotic signaling pathways. Treatment with TRAIL, tumor necrosis factor-related apoptosis-inducing ligand, in association with numerous anticancer compounds was stated to be effective in removing CSCs (Fox et al., 2010). Co-treatment of cisplatin was designated to be very important effective in decreasing triple negative breast CSCs via Wnt signaling inhibition as well as apoptosis induction (Yin et al., 2011a, b). Similarly, TRAIL treatment with daunorubicin or cytarabine has been revealed to reduce acute myeloid progenitor’s growth (Plasilova et al., 2002). Additionally, Bortezomib, a proteasome inhibitor, along with TRAIL, was exposed to trigger GBM stem cells apoptosis (Unterkircher et al., 2011). Besides, MSCs express TRAIL via lentiviral vector transduction was used to stimulate apoptosis in lung and squamous CSC population in holding tumors of nude mice (Loebinger et al., 2010). NF-κB, nuclear factor kappa-light-chain-enhancer of activated B cells, in another apoptosis inducing target of CSCs. Oxyresveratrol, derived from the *Morus alba* root extract, has been shown to cause accumulation ROS and induction of autophagic, as well as apoptotic, cell death through the FOXO-caspase 3-mediated pathway in neuroblastoma cells (Kwon et al., 2015; Rahman et al., 2017). Human neuroblastoma induced by angelicin and gambogic acid shown apoptosis cell death (Rahman et al., 2012b, 2013). Predominantly, NF-κB prevents apoptosis as a result stimulates cell proliferation, tumor progression, angiogenesis inflammation, as well as metastasis (Park and Hong, 2016). However, NF-κB pharmacologically inhibited via parthenolide, pyrrolidinedithiocarbamate as well as its analog diethyldithiocarbamate specially aim to control breast CSCs (Zhou et al., 2008). These observations emphasize that NF-κB is an important to conserve and maintain tumor-initiating CSCs survival (Zhou et al., 2008). Furthermore, NF-κB inhibition with MG-132, proteasome inhibitor, along with idarubicin stimulated apoptosis of leukemic stem cells with significantly lesser that in normal hematopoietic stem cells (Guzman et al., 2002). Natural plant extracts from *Melandrium firmum*, *Dioscorea nipponica* Makino, and *Saussurea lappa* Clarke has been shown to have anti-proliferative and apoptotic effects in neuroblastoma cells (Rahman et al., 2013, 2014, 2015). Therefore, apoptosis signaling to CSC control in preclinical examination to propose the opportunity to eradicate cancers treatment.

## Therapeutic Aspects of Autophagy for the Cancer Stem Cells Treatment

One of the most important point is the drug resistance that outcomes in tumor relapse remains a main impediment to development survival of cancer patient. Besides, drug resistance might be owing to the existence of CSCs. Table 1 premises the various studies that have examined natural compounds that possible target CSCs via autophagy regulation. Targeting signaling of these compounds comprises inhibitors of Akt, mTOR, tyrosine kinases, HDAC, proteasome, IL6 signaling, farnesyl transferase, hypoxia, Notch, Wnt signaling, as well as hedgehog signaling. However, depending on the kind of cancer in addition the compound, induction or inhibition of autophagy has been considered for therapeutic target in CSCs treatment. For complete eradication of cancer, it might be essential to target non-CSC and CSCs populations. For instance, in one study shown that CQ, autophagy inhibitor, targets pancreatic CSCs through CXCR4 inhibition as well as independently of autophagy in hedgehog signaling (Balic et al., 2014). One of the few drugs, for example, metformin (Hirsch et al., 2009) and salinomycin (Gupta et al., 2009b) have been recognized to target CSCs control *in vitro* as well as *in vivo* effectively to eliminate CSCs alone or in combination by conventional chemotherapeutic drugs (Vidal et al., 2014). Pharmacological inhibitor such as PTC-209 and BMI-1 also revealed to successfully prevent self-renewal of CSC *in vitro* in addition to efficiently block tumor growth and progression of mouse xenografts model (Kreso et al., 2014).

Based on current studies, a combination of modulators of autophagy and standard chemotherapeutic agents could represent an effective anticancer treatment strategy. However, there are numerous bottlenecks in the development of an effective anticancer treatment targeting autophagy, as its reaction differs according to the origin and type of cell, stimulus, and stress conditions. However, the effects of autophagy in the tumor microenvironment should be validated in further studies. Furthermore, novel as well as reliable approaches for regulating autophagy in clinical models need to be established. Accordingly, an understanding of the functional role of autophagy in the response to therapies might contribute to overcoming the resistance to chemotherapy and sensitizing CSCs to anticancer treatments. There is growing evidence that autophagy is a promising target for stabilizing CSC invasion and aggressiveness. It is also essential to consider that CQ in addition to its derivatives, such as hydroxychloroquine, have been evaluated in numerous clinical trials and may be effective in combination with traditional anticancer therapies (Chen and Chan, 2017). Most importantly, CSC heterogeneity in addition to patient-specificity presents an increase in complexity that has not been previously understood. We are far from establishing unique and well-known combinations of drugs able to eliminate CSCs or, at minimum, to prevent and block their proliferation and growth. However, global efforts are focused on the incorporation of recent discoveries into novel therapeutic approaches. It has been propose that both the inhibition and activation of autophagy are targets for sensitizing and maintaining CSCs. The outcomes of CSC management and the effectiveness of CQ in anti-CSC therapies might depend on tumor growth properties, and autophagy-mediated CSC processes may be important determinants. Considerably more precise and stronger lysosome blockers than CQ are being identified. These drugs provide an opportunity to develop comprehensive combinations of treatments for CSCs, including pepstatin A (which blocks cathepsins D and E), E64d (blocker of cathepsins B, H, and L), or concanamycin A (a selective blocker of V-ATPase, which inhibits fusion between lysosomes as well as endosome acidification) (Yang et al., 2013). Conversely, the inhibition of autophagosome elimination and degradation does not disturb cargo sequestration and autophagosome maturation or formation. Therefore, drugs that are effective against the early phases of autophagy, such as a VPS34 inhibitor (SAR405) or PIK-III or ULK1 inhibitor (MRT68921), might provide better results with respect to controlling CSCs (Dowdle et al., 2014; Petherick et al., 2015). Additional examinations of the molecular mechanism and mode of action of CSC specific drugs along with non-CSC plasticity and role of autophagy are required before vastly effective treatments might be well-known for patients in all aspects of cancer.

## Clinical Implications of Autophagy in Cancer Stem Cell Control

Therefore, regulatory factors along with molecular and cellular mechanisms by which autophagy exerts a functional role in CSCs are essential for the development of effective and safe antitumor approaches. The combined use of autophagy blockers/activators with chemotherapeutic drugs, the examination of the role of autophagy in the immune response, as well as virotherapy might be critical to develop novel approaches against CSCs as alternatives to conventional treatment. Furthermore, it is important to consider that solid tumors generally develop in environments with low oxygen levels, creating a niche to defend against CSCs, with additional destructive effects as well as resistant to cell death. Additional studies are therefore required to characterize the functions of autophagy in interference among stromal cells, adaptive immune cells, endothelial cells, and tumor-infiltrating innate immune cells. Notably, these kinds of cells might have different requirements for autophagy, making it difficult to develop autophagic therapies for CSCs. Further studies are also required for the development of novel and reliable approaches for measuring autophagic flux activation in patient samples in clinically. Undoubtedly, the sequestration of CSCs from the blood is an effective technique for observing autophagy. In future, RNA sequencing, a groundbreaking tool for transcriptome analyses, is promising for understanding the molecular determinants of the initiation and stimulation of autophagy.

Additionally, recent results enable us to identify novel and interesting scenarios for the modulation and control of autophagy in the microenvironment adjacent to CSCs. Malignant cells stimulate autophagy in the microenvironment in addition to distal tissues for the maintenance of self-growth and proliferation by enhancing the accessibility of reprocessed nutrients. However, blocking or reducing autophagy in the tumor yields adequate results with respect to tumor invasion and development, whereas the prevention of autophagy by the oral administration of CQ results in an additional visible decline in tumor progression, proliferation, as well as invasion (Katheder et al., 2017). Therefore, metabolic crosstalk between non-CSCs and CSCs as well as other cancer-associated fibroblasts (CAFs) establishes additional relationships (Yoshida, 2017). As a result, it could be hypothesized that targeting non-CSCs as well as CAFs by blocking autophagy might decrease nutrient accessibility and might negatively influence the intrinsic resistance of CSCs and mechanisms against chemotherapeutic strategies. Nonetheless, interventions that hinder autophagy might have unintended adverse side effects related to immune surveillance. Recent studies have shown that caloric restriction and fasting-dependent autophagy stimulation affect anticancer immune surveillance, thereby promoting tumor cell growth properties as well as improvements in responses to chemotherapy (Pietrocola et al., 2016).

## Concluding Remarks

Recent studies suggest that enhancing CSC subpopulations can improve the outcomes of cancer therapy and prevent tumor recurrence after chemotherapy. The existence of CSCs in tumors expressively contributes to chemo- and radioresistance of tumors as well as metastasis. Autophagy eradicates and reprocesses unwanted cellular components. It promotes resistance to anticancer therapy. An easy method for controlling CSCs via autophagy has not been developed. Many investigators believe that autophagy functions to maintain CSC stemness, and thereby results in the failure of anticancer treatment. Autophagy may contribute to the loss of stemness in certain CSCs. Consequently, autophagy could serve as a prospective target for reducing the resistance of CSCs to anticancer treatment. Reducing the autophagic flux may not only improve chemotherapeutic properties, but may also deliver additional nutrient sources for cancer cells. The optimal doses of autophagy inhibitors have not been determined. Studies are based on cell lines as well as animal models. Additional clinical studies are required to verify the results of these investigations. Various additional factors must be considered before clinical applications. Nonetheless, the treatment of CSCs with autophagy modulators is a potentially beneficial approach for anticancer treatment. Therefore, it is crucial to perform additional experiments aimed at targeting autophagy signaling for controlling CSCs. More specifically, the biological effects of autophagy are still not fully understood. However, the effects of autophagy might depend on several factors, such as the cell type, stimulus, and microenvironment. Thus, an exciting and novel modulator of autophagy that is effective and safe is required for the treatment of CSCs.

## Author Contributions

This work was collaboration among all of the authors. MAR and SS designed outlines and wrote the draft of the manuscript. MSR prepared the figures. MJU and MSU wrote the initial draft of the manuscript. M-GP reviewed the manuscript. HR and S-GC proposed the original idea and reviewed the scientific contents described in the manuscript. All authors read and approved the final submitted version of the manuscript.

## Conflict of Interest

The authors declare that the research was conducted in the absence of any commercial or financial relationships that could be construed as a potential conflict of interest.
